# Thromboembolic events with olanzapine: a systematic review integrating meta-analysis and FAERS database

**DOI:** 10.3389/fcvm.2026.1710507

**Published:** 2026-02-11

**Authors:** XueSong Zhang, SiMan Sun, XiaoYu Fan, ShuShu Guo, WanFang Li, Chuan Wang, Jia Xu, ChongZe Chen, HongTao Jin

**Affiliations:** 1 School of Pharmacy, Shaanxi University of Chinese Medicine, Xianyang, China; 2 New Drug Safety Evaluation Center, Institute of Materia Medica, Chinese Academy of Medical Sciences & Peking Union Medical College, Beijing, China; 3 College of Life Sciences and Biopharmaceuticals, Shenyang Pharmaceutical University, Shenyang, China; 4 Beijing Union-Genius Pharmaceutical Technology Development Co., Ltd., Beijing, China; 5 Department of Dermatology, Beijing Hospital of Traditional Chinese Medicine, Capital Medical University, Beijing, China; 6 Department of Pharmacy, Fuzhou Changle People’s District Hospital, Fuzhou, China; 7 Beijing Key Laboratory of Key Technologies for Preclinical Research and Development of Innovative Drugs in Pharmacokinetics and Pharmacodynamics, Beijing, China

**Keywords:** FAERS database, meta-analysis, olanzapine, pulmonary embolism, venous thromboembolism

## Abstract

**Background:**

Olanzapine is an atypical antipsychotic used to treat schizophrenia and manic episodes. Its potential thromboembolic risk has been reported, but the evidence remains controversial. This study aimed to comprehensively evaluate the association between olanzapine and pulmonary embolism (PE) and venous thromboembolism (VTE).

**Methods:**

This study combined meta-analysis and signal mining from the FDA Adverse Event Reporting System (FAERS) database to assess the association between olanzapine and pulmonary embolism (PE) and venous thromboembolism (VTE).

**Results:**

From 55,905 olanzapine-related adverse event reports in the Faers database, 1,233 significant signals were identified, including serious adverse events not fully documented on the drug label, such as pulmonary embolism and venous embolism. A meta-analysis of eight studies showed that olanzapine use significantly increased the risk of VTE and pulmonary embolism (OR = 2.07, 95% CI: 1.37–3.14, *P* = 0.0006).

**Conclusion:**

These findings suggest that olanzapine is associated with an increased risk of thromboembolic events; therefore, enhanced clinical surveillance and further investigation into its safety are necessary.

**Systematic Review Registration:**

https://www.crd.york.ac.uk/prospero/, identifier CRD420251003254.

## 1 Introduction

Olanzapine is an atypical antipsychotic drug belonging to the thienobenzodiazepine class. It exhibits high binding affinity for various neurotransmitter receptors, including dopamine (DA), serotonin (5-HT), histamine (H1), adrenergic (*α*1), and muscarinic (M) receptors. Olanzapine was first introduced in Switzerland in 1996 and subsequently in China in 1999 (1). Clinically, olanzapine is primarily used to treat schizophrenia and acute manic or mixed episodes associated with bipolar I disorder. It can also be used as monotherapy or adjunctive therapy to effectively treat depressive episodes of bipolar I disorder and for maintenance therapy. Furthermore, due to its potent antiemetic properties through antagonism of dopamine and serotonin receptors, olanzapine has been investigated and used off-label for the prevention of chemotherapy-induced nausea and vomiting (2, 3).

Thromboembolic events, particularly pulmonary embolism (PE) and venous thromboembolism (VTE), are potentially life-threatening serious adverse events. PE is a pathological condition caused by endogenous or exogenous emboli obstructing the pulmonary artery and its branches, primarily manifesting as dyspnea, chest pain, and hypoxemia. In severe cases, it can be fatal (4). Most cases are acute (5), while a few have a chronic course (6), often accompanied by signs and symptoms related to deep vein thrombosis (DVT) in the lower extremities (7).VTE is a pathological condition caused by the formation of thrombi that obstruct venous blood flow. It primarily includes DVT, which involves clot formation in the deep veins, and PE, which occurs when a thrombus dislodges and travels to the lungs, resulting in occlusion of the pulmonary artery (8). Furthermore, the incidence of VTE increases exponentially with age, with a markedly elevated risk observed in individuals over 40 years of age (9).

The association between conventional antipsychotic drugs and VTE was first reported in the 1950s (10). In 1997, Walker et al. reported the association between atypical antipsychotic drugs and thrombosis, finding that the use of these drugs increased the risk of PE by five times (11). Two case reports documented pulmonary embolism and venous thromboembolism events that occurred after the initiation of olanzapine treatment (12, 13). A retrospective study conducted by the Becksa County Medical Examiner's Office in 2008 found a temporal association between olanzapine use and pulmonary embolism in four antipsychotic drug-related deaths that occurred between 1998 and 2005 (14). Subsequent studies have shown that olanzapine may significantly increase the incidence of venous thromboembolism and pulmonary embolism, particularly in older patients (15).

Studies have shown that olanzapine can induce metabolic disorders, including hyperglycemia, hyperleptinemia, and dyslipidemia (16). Its thrombotic mechanisms involve multiple pathways, including promoting platelet aggregation by activating serotonin receptors and inducing venous congestion through alpha receptor-mediated hypotension (17). Olanzapine can also impair vascular endothelial function by increasing inflammatory markers such as C-reactive protein (CRP) and interleukin-6 (IL-6) (18). In addition, olanzapine can stimulate the production of prolactin-mediated antiphospholipid antibodies (19) or induce acetyl-CoA carboxylase phosphorylation, thereby affecting vascular function (20). These mechanisms together constitute the pathological basis of olanzapine-related thrombosis.

Traditional antipsychotics (chlorpromazine) have been shown to be associated with an increased risk of VTE, but the conclusions regarding atypical antipsychotics (olanzapine) are still unclear (21). Although previous systematic reviews have explored the relationship between antipsychotics and VTE and PE, there is still a lack of specific analyses of olanzapine (22). To address this gap, we conducted a systematic review and meta-analysis, integrated the observational evidence from existing cohort studies and case-control studies, and calculated the combined effect value (OR) and its confidence interval (CI) through strict data extraction, quality assessment (Newcastle-Ottawa scale) and statistical analysis (Review Manager 5.4 and Stata 16 software) to quantitatively evaluate the strength of the association between the use of olanzapine and the risk of PE and VTE. At the same time, we conducted pharmacovigilance analysis based on the FDA Adverse Event Reporting System (FAERS) database (23, 24) to provide more comprehensive and accurate evidence for the assessment of this clinical safety issue.

## 2 Materials and methods

This review was registered with PROSPERO (International Prospective Register of Systematic Reviews; ID: CRD420251003254), and the meta-analysis was conducted and reported in strict accordance with the 27 items outlined in the PRISMA (Preferred Reporting Items for Systematic Reviews and Meta-Analyses) checklist (25). Literature screening, study selection, data extraction, and quality assessment were independently performed by two researchers. Any discrepancies were resolved through discussion or, if necessary, consultation with a third reviewer.

### 2.1 FAERS database

#### 2.1.1 FAERS database mining

FAERS is a publicly accessible post-marketing pharmacovigilance database that compiles ADE reports from a wide range of sources, including healthcare professionals, patients, pharmaceutical companies, and legal representatives. The database receives global ADE submissions and is updated on a quarterly basis. It contains detailed information on reporting sources, demographic and administrative data, medication usage, indication or diagnosis, treatment start and end dates, reported adverse events, patient outcomes, and cases deemed invalid. In this study, all available data from FAERS across 35 quarters—from the first quarter of 2016 to the third quarter of 2024—were extracted and imported into RStudio for data cleaning and subsequent analysis.

#### 2.1.2 Data extraction and screening

This study focused on the safety of olanzapine monotherapy; therefore, the “role_cod” field in the extracted reports was limited to the primary suspect drug (PS). Both the trade name Zyprexa and the generic name olanzapine were used as search terms, and only ADE reports in which olanzapine was identified as the primary suspected drug were included. ADE terms were coded using the Preferred Term (PT) level of the Medical Dictionary for Regulatory Activities (MedDRA®) version 26.0, as defined by the International Council for Harmonisation of Technical Requirements for Pharmaceuticals for Human Use (ICH). PTs were further classified, organized, and quantified based on the System Organ Class (SOC) categories in MedDRA (26). To eliminate duplicate records, reports were sorted by CASEID, FDA_DT, and PRIMARYID. For multiple entries with the same CASEID, the report with the latest FDA_DT was retained; if both CASEID and FDA_DT were identical, the report with the highest PRIMARYID was selected (27). In addition, clinical characteristics of patients who experienced adverse events following olanzapine use—including age, sex, reporter type, reporting region, body weight, and patient outcomes—were also collected.

#### 2.1.3 Signal mining algorithm

This study employed a 2  ×  2 contingency table based on the odds imbalance method (Table 1) and applied both the reporting odds ratio (ROR) (28) and the proportional reporting ratio (PRR) to detect ADE signals associated with olanzapine, thereby enhancing the robustness of signal detection results (29). The ROR offers an advantage in adjusting for potential bias arising from low-frequency event reporting, while the PRR demonstrates relatively higher specificity compared to ROR. The formulas and signal detection thresholds for both algorithms are presented in Table 2. Statistical analyses were conducted using R software. Higher ROR or PRR values reflect greater signal strength, indicating a stronger association between olanzapine and the reported ADE.

**Table 1:** Four-grid table of ratio imbalance measurement method.

Drug	Target Event	Other events	Total
Target Drugs	a	b	a + b
Other medicines	c	d	c + d
Total	a + c	b + d	a + b + c + d

a: The number of reports that record both the target drug and its corresponding specific adverse events; b: The number of reports of other atypical adverse events caused by the target drug; c: The number of reports of similar adverse events caused by non-target drugs; d: The number of reports containing non-target drugs and their related adverse events.

**Table 2:** Calculation formula and signal generation standard of ratio imbalance method.

Method	Formula	Signal generation standards
ROR	ROR=ad/bc	*N* ≥ 3, ROR 95%CI > 1
	95%CI = eln(ROR) ± 1.96(1/a + 1/b + 1/c + 1/d)^0.5	
PRR	PRR = a(c + d)/c/(a + b)	*N* ≥ 3, PRR≥2, *χ*^2^ ≥ 4
	χ2 = [(ad-bc)^2](a + b + c + d)/[(a + b)(c + d)(a + c) (b + d)]	

a: The number of reports that record both the target drug and its corresponding specific adverse events; b: The number of reports of other atypical adverse events caused by the target drug; c: The number of reports of similar adverse events caused by non-target drugs; d: The number of reports containing non-target drugs and their related adverse events; N: The number of reports. PRR, Proportional Reporting Ratio; ROR, Reporting Odds Ratio.

### 2.2 Meta-analysis

#### 2.2.1 Search strategy

The literature search strategy was based on a computer system to search for relevant literature published before January 2025 in PubMed, Web of Science, Embase, and SCOPUS databases. The language of literature search was set to English. A combination of subject terms and free terms were used in all searches. The subject headings included ‘Olanzapine’, ‘Pulmonary embolism’, ‘Venous thromboembolism’, and the free terms included ‘2- Methyl- 4- (4- methyl- 1- piperazinyl)-10H-thieno(2,3-b) (1,5) benzodiazepine’, ‘Zyprexa’, ‘Olanzapine Pamoate’, ‘Zolafren’ and ‘pulmonary embolisms’, ‘Pulmonary Thromboembolisms’, ‘Pulmonary Thromboembolism’. For detailed search strategies, see Supplementary Figure S1.

#### 2.2.2 Inclusion and exclusion criteria

This study followed the PICOS (Population, Intervention, Comparison, Outcome, Study design) framework (30), and applied strict inclusion and exclusion criteria for literature selection. The population included adult patients (≥18 years old) with a confirmed diagnosis of schizophrenia or mania. The intervention of interest was treatment with olanzapine, regardless of dosage, treatment duration, or route of administration (e.g., oral or injectable), either as monotherapy or in combination with other agents. The comparison group consisted of similar patients who did not receive olanzapine but were treated with other antipsychotic medications, as well as healthy individuals from the same patient source. Eligible study designs included observational studies (e.g., cohort studies and case-control studies) and randomized controlled trials (RCTs). Only studies published in English were considered. The primary outcome was the incidence of clinically confirmed VTE or PE in the olanzapine group vs. the control group. In addition, studies were required to report effect estimates such as relative risk (RR), odds ratio (OR), and their corresponding 95% CIs, or other extractable measures of association.

The following types of literature were excluded from this study: (1) case reports, cross-sectional studies, and experimental studies (including *in vitro* and animal experiments); (2) review articles, conference abstracts, studies without control groups, editorial letters, and studies with missing or invalid data; (3) publications in which the study population, intervention, or outcome measures did not meet the inclusion criteria; and (4) studies for which the full text was not available.

#### 2.2.3 Literature screening and data extraction

An initial screening was conducted by two investigators who independently assessed the titles and abstracts of all identified publications. After excluding irrelevant studies and those for which the full text was unavailable, the remaining articles were reviewed in full to determine eligibility based on the predefined criteria. In cases of disagreement, a third reviewer was consulted to reach a consensus. For studies that met the inclusion criteria, the following information was extracted: study title, first author, year of publication, data source (country), sex distribution of the study population, age range, study design, number of participants in the intervention and control groups, treatment duration, outcome measures, and study quality.

#### 2.2.4 Quality assessment

Two researchers independently evaluated the quality of the included literature, and any disagreements were resolved through discussion by a third researcher. The NOS was used to assess the methodological quality of cohort and case-control studies. Studies with NOS scores below the average were defined as low quality (≤3 points), studies with NOS scores around the average were defined as moderate quality (4–6 points), and studies with NOS scores equal to or above the average were defined as high quality (≥7 points).

#### 2.2.5 Data analysis

Statistical analyses were performed using RevMan, which was also used to generate forest plots. Adjusted ORs, RRs, and their corresponding 95% CIs reported in the included studies were analyzed. To assess heterogeneity among study results, the *I²* statistic was calculated. Heterogeneity was interpreted based on the *I²* value: if no significant heterogeneity was detected (*P* ≥ 0.1, *I²* ≤ 50%), a fixed-effects model was applied for meta-analysis (31); otherwise, if significant heterogeneity was present (*P* < 0.1, *I²* > 50%), a random-effects model was used, and regression analysis was performed to identify the source of heterogeneity. Sensitivity and subgroup analyses were performed to evaluate the stability of the results (32). Publication bias was assessed using funnel plots and Egger's test, conducted in Stata version 16. For Egger's test, a significance level of *α* = 0.05 was used, where *P* < 0.05 indicates the presence of publication bias, and *P* ≥ 0.05 indicates no significant publication bias.

## 3 Results

### 3.1 FAERS database mining

#### 3.1.1 Basic information of olanzapine ADE report

**Figure 1:**
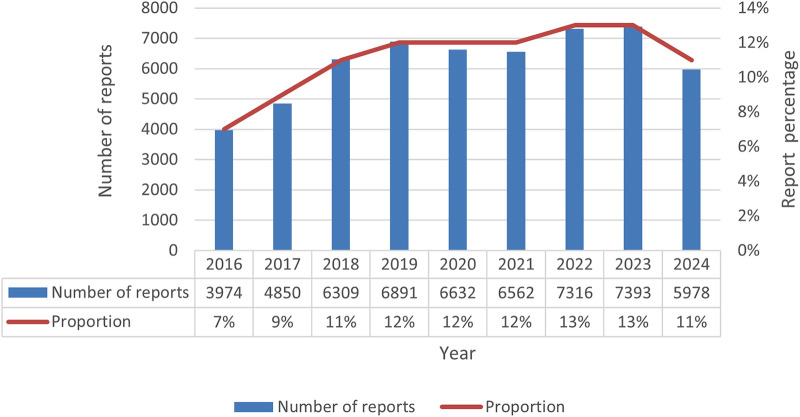
Number of olanzapine adverse event reports by year.

A total of 254,126 olanzapine ADE reports were extracted from the database. After deleting duplicate data and excluding ADEs that could not be assessed, a total of 55,905 ADE reports of olanzapine were screened. The data showed that the number of reports from male patients (45.1%) exceeded that from female patients (43.8%). This difference may be related to the anticholinergic effect of olanzapine, which can increase prolactin concentrations and thus cause breast enlargement and lactation in men (33). A large amount of patient weight information was missing in the reports collected in this study, which may have caused some interference in the assessment of weight-related adverse events (such as weight gain). Among reports with clear age data, the age group with the largest number of reports was 18–64 years old. The reports are mainly sourced from the United States, Canada and the United Kingdom. The majority of reporters were doctors (29.8%). From the perspective of patient outcomes, the main outcomes were hospitalization (2,0471, 36.6%), death (6,375, 11.4%), life-threatening (4,280, 7.7%), disability (960, 1.7%), and other serious events (15,779, 28.2%). This suggests the importance of monitoring olanzapine-related ADEs, as detailed in Table 3. Judging from the distribution of reporting time (Figure 1), the number of reports showed a gradually increasing trend, among which the largest number of reports was submitted in 2,023 (7,393 reports), accounting for 13%. The number of reports was the lowest in 2016 (3,974), accounting for 7%, and increased by 10.3% per year from 2016 to 2023.

**Table 3:** Basic information of adverse event reports related to olanzapine.

Basic Information	Classification	Number of reports (Proportion/%)
Gender	Female	24,499 (43.8%)
	Male	25,196 (45.1%)
	Unknown	6,210 (11.1%)
Weight	<50 kg	1,738 (3.1%)
	50∼100 kg	10,510 (18.8%)
	>100 kg	1,786 (3.2%)
	Unknown	41,871 (74.9%)
Age	18∼40	13,341 (23.9%）
	41∼64	16,306 (29.2%）
	65∼85	8,203 (14.7%)
	≥86	906 (1.6%)
	Unknown	14,433 (25.8%)
Reporter	Doctor	16,652 (29.8%)
	Consumer	14,325 (25.6%)
	Other health professionals	6,996 (12.5%)
	Pharmacist	4,648 (8.3%)
	lawyer	298 (0.5%)
	Unknown	1,008 (1.8%)
Outcome	Hospitalization	20,471 (36.6%)
	Other serious incidents	15,779 (28.2%)
	Death	6,375 (11.4%)
	Life-threatening	4,280 (7.7%)
	Disability	960 (1.7%)
	Congenital malformations	260 (0.5%)
	Intervention is needed to prevent permanent damage	18 (0.0%)
	Unknown	7,762 (13.9%)
Reporting Country	USA	16,580 (29.7%)
	Canada	6,177 (11.0%)
	U.K.	5,706 (10.2%)
	France	4,164 (7.4%)
	Italy	3,205 (5.7%)
	Germany	2,287 (4.1%)
	Japan	2,248 (4.0%)
	China	1,003 (1.8%)

#### 3.1.2 Distribution of ADE signals in different SOCs

According to the adverse event signal evaluation criteria, a total of 1,261 adverse drug event signals with olanzapine as the primary suspected drug were discovered. After excluding two SOCs (product issues and social environment) that were not directly related to olanzapine-related adverse events, there were 1,233 valid signals remaining, covering 25 SOCs.

According to the ranking of adverse event reports, the top 10 SOCs are mental illness, various neurological diseases, various examinations, various injuries, etc. (Table 4). Among them, the SOCs involved in adverse events not recorded in the instructions include mental illness (suicidal behavior), various examinations (prolonged QT interval of electrocardiogram, increased blood creatine phosphokinase), metabolic and nutritional diseases (hyponatremia), heart diseases (myocarditis, cardiomyopathy), blood and lymphatic system diseases (febrile neutropenia, leukocytosis), respiratory system, chest and mediastinal diseases (pulmonary embolism).

**Table 4:** Top 10 SOCs with the highest number of olanzapine adverse event reports.

SOC	Number of PTs	Adverse event reports	PT (top 5 Cases)
Psychiatric	251	34,755	Suicidal behavior* (2,030), Confusional state (1,717), Substance abuse (1,605), Schizophrenia (1,595), Psychotic disorder (1,496)
Various neurological diseases	186	24,807	Somnolence (2,294), Sedation (1,743), Neuroleptic malignant syndrome (1,425), Tremor (1,401), Akathisia (1,141)
Various inspections	191	12,084	Weight gain (2,718), prolonged QT interval on electrocardiogram* (1,424), increased blood creatine phosphokinase* (538), abnormal blood prolactin (529), decreased neutrophil count (492)
Various injuries, poisoning and procedural complications	49	6,485	Toxicity of various preparations (2,993), intentional overdose (1,634), medication errors (340), prescription overdose (252), intentional poisoning (250)
Systemic diseases and administration site reactions	41	5,696	Drug interactions (2,567), treatment noncompliance (839), hypothermia (349), drug resistance (298), hyperthermia (206)
Metabolic and nutritional diseases	53	4,934	Hyponatremia* (743), Diabetes (547), Metabolic disorders (515), Obesity (475), Increased appetite (440)
Heart disease	61	4,637	Tachycardia (1,288), Myocarditis* (526), Cardiorespiratory arrest (417), Sinus tachycardia (317), Cardiomyopathy* (184)
Blood and lymphatic system diseases	24	4,070	Neutropenia (1,310), febrile neutropenia* (998), leukopenia (666), agranulocytosis (304), leukocytosis* (204)
Respiratory, thoracic and mediastinal diseases	47	2,843	Pulmonary embolism* (718), respiratory depression (306), respiratory distress (197), respiratory arrest (193), aspiration (160)
Reproductive system and breast diseases	26	2,021	Sexual dysfunction (678), male breast development (386), erectile dysfunction (195), priapism (146), amenorrhea (143)

* Adverse events not listed in the instructions.

PT, Preferred Term; SOC, System Organ Class.

#### 3.1.3 Analysis of olanzapine-related ADE signal intensity

ADE signal strength was screened using the reporting ROR and PRR methods, resulting in the identification of 1,233 eligible preferred terms (PTs) with a cumulative total of 113,271 reports. After excluding PTs that were clearly unrelated to the drug, product issues, and olanzapine's indications, the top 50 PTs were selected based on the number of reported cases (Table 5). The top five most frequently reported symptoms were weight gain, drowsiness, suicidal behavior, sedation, and confusion. To assess whether these signals were included in the drug label, the top 50 adverse events were compared with the latest olanzapine prescribing information (updated in 2025) from the FDA Label on the official FDA website, supplemented by a review of primary literature on related adverse events to verify their inclusion (34). The comparison revealed that 39 of the top 50 PTs were explicitly mentioned in the FDA label, demonstrating high consistency with the adverse events identified in this study and supporting the credibility and clinical relevance of the mined signals. The remaining 11 PTs were not mentioned in the label and may represent potential new signals; these included suicidal behavior, prolonged QT interval on electrocardiogram, febrile neutropenia, delirium, hyponatremia, pulmonary embolism, attention disorder, catatonia, abnormal blood prolactin levels, myocarditis, infectious aspiration pneumonia, and excessive salivation, indicating that these signals warrant further investigation and validation.

**Table 5:** Top 50 PTs with the highest number of adverse events associated with olanzapine.

No.	PT	Number of cases	ROR (95%CI)	PRR (χ^2^)
1	Weight gain	2,718	3.15 (3.03–3.27)	3.13 (3,862.08)
2	Lethargy	2,294	2.96 (2.84–3.09)	2.95 (2,897.14)
3	Committing suicidal behavior*	2,030	9.84 (9.41–10.3)	9.77 (14,956.21)
4	Sedation	1,743	21.55 (20.49–22.67)	21.41 (29,419.86)
5	Confusion	1,717	2.84 (2.71–2.98)	2.83 (1,996.53)
6	Neuroleptic malignant syndrome	1,425	46.62 (43.91–49.51)	46.37 (47,501.45)
7	Electrocardiogram (ECG) QT interval prolongation*	1,424	9.82 (9.3–10.36)	9.77 (10,480.29)
8	Tremor	1,401	2.32 (2.2–2.44)	2.31 (1,027.92)
9	Suicidal thoughts	1,323	4.19 (3.97–4.43)	4.18 (3,107.04)
10	Neutropenia	1,310	2.13 (2.02–2.25)	2.13 (771.12)
11	Tachycardia	1,288	3.92 (3.71–4.14)	3.9 (2,707.68)
12	Suicide	1,243	4.58 (4.32–4.84)	4.56 (3,347.63)
13	Akathisia	1,141	28.1 (26.36–29.94)	27.97 (24,731.18)
14	Extrapyramidal disorders	1,106	30.28 (28.37–32.32)	30.15 (25,644.76)
15	Deliberate self-harm	1,004	10.67 (10–11.38)	10.63 (8,142.73)
16	Febrile neutropenia*	998	3.64 (3.41–3.87)	3.62 (1,851.04)
17	Movement disorders	997	7.28 (6.83–7.76)	7.26 (5,117.86)
18	Dystonia	954	24.18 (22.57–25.91)	24.1 (18,015.5)
19	Coma	939	5.45 (5.11–5.82)	5.44 (3,275.91)
20	Decreased level of consciousness	916	6.59 (6.16–7.04)	6.57 (4,130.38)
21	Sleep	900	14.98 (13.98–16.04)	14.93 (10,567.68)
22	delirium*	822	6.45 (6.01–6.92)	6.43 (3,607.71)
23	Parkinson's disease	746	24.86 (22.99–26.87)	24.79 (14,465.81)
24	Hyponatremia*	743	3.54 (3.29–3.81)	3.54 (1,319.29)
25	Pulmonary embolism*	718	2.45 (2.28–2.64)	2.45 (604.72)
26	Restlessness	693	5.28 (4.9–5.7)	5.27 (2,313.2)
27	Sexual dysfunction	678	19.02 (17.55–20.61)	18.97 (10,162.87)
28	Leukopenia	666	3.45 (3.19–3.72)	3.44 (1,126.93)
29	Rhabdomyolysis	630	4.71 (4.35–5.1)	4.7 (1,774.67)
30	Attention deficit disorder*	591	2.85 (2.62–3.09)	2.84 (692.45)
31	Cognitive impairment	587	3.13 (2.88–3.39)	3.12 (827.94)
32	Catatonia*	584	35.25 (32.19–38.6)	35.17 (15,490.87)
33	Diabetes	547	2.11 (1.94–2.3)	2.11 (314.62)
34	Mental damage	541	6.16 (5.65–6.71)	6.15 (2,234.58)
35	Elevated blood creatine phosphokinase	538	6.56 (6.02–7.15)	6.55 (2,416.84)
36	Dysarthria	533	4.15 (3.81–4.53)	4.15 (1,235.94)
37	Abnormal blood prolactin*	529	406.96 (343.86–481.64)	406.12 (54,723.54)
38	Myocarditis*	526	11.41 (10.44–12.47)	11.39 (4,609.25)
39	Tardive dyskinesia	523	13 (11.88–14.22)	12.97 (5,289.94)
40	Abnormal behavior	520	4.49 (4.11–4.9)	4.48 (1,362.99)
41	Metabolic disorders	515	33.09 (30.06–36.43)	33.02 (12,936.17)
42	Personality changes	501	17.69 (16.12–19.42)	17.66 (6,991)
43	Infectious aspiration pneumonia*	498	5.11 (4.67–5.59)	5.1 (1,584.35)
44	Decreased neutrophil count	492	2.87 (2.62–3.13)	2.86 (584.56)
45	Speech disorders	484	2.51 (2.29–2.74)	2.5 (430.15)
46	Irritability	476	2.19 (2–2.39)	2.18 (301.34)
47	Obesity	475	7.67 (7–8.42)	7.66 (2,608.31)
48	Serotonin syndrome	475	6.8 (6.2–7.46)	6.79 (2,236.97)
49	Excessive salivation*	472	13.47 (12.26–14.81)	13.45 (4,961.8)
50	Hyperprolactinemia	468	16.34 (14.85–17.98)	16.31 (6,023.78)

* Adverse events not listed in the instructions.

PT, Preferred Term; PRR, Proportional Reporting Ratio; ROR, Reporting Odds Ratio.

#### 3.1.4 Analysis of ADE signal intensity in olanzapine-related embolism and thrombosis

After a secondary search of embolism and thrombosis, it was found that there were 9 PTs related to embolism risk associated with olanzapine, with 843 cases. The top three PTs in terms of case numbers were pulmonary embolism [718 cases, ROR (95% CI): 2.45 (2.28, 2.64), PRR (*χ*2): 2.45 (604.72)], venous embolism [73 cases, ROR (95% CI): 5.67 (4.49, 7.17), PRR (*χ*2): 5.67 (270.03)], and embolism [28 cases, ROR (95% CI): 0.94 (0.64, 1.36), PRR (*χ*2): 0.94 (0.12)]. Detailed data are shown in Table 6.

**Table 6:** PTs with signal intensity of thrombotic and embolic risk in olanzapine.

No.	PT	Number of cases	ROR (95%CI)	PRR (χ^2^)
1	Pulmonary embolism*	718	2.45 (2.28–2.64)	2.45 (604.72)
2	Venous embolism*	73	5.67 (4.49–7.17)	5.67 (270.03)
3	Emboembolism*	28	0.94 (0.64–1.36)	0.94 (0.12)
4	Paradoxical embolism*	10	35.83 (17.89–71.77)	35.83 (269.45)
5	Arterial embolism*	8	2.5 (1.24–5.03)	2.5 (7.08)
6	Peripheral embolism*	2	0.6 (0.15–2.41)	0.6 (0.53)
7	Air embolism*	2	1.27 (0.32–5.11)	1.27 (0.11)
8	Cerebral arterial embolism*	1	0.25 (0.04–1.79)	0.25 (2.22)
9	Cerebral air embolism*	1	17.47 (2.18–139.65)	17.47 (13.8)

* Adverse events not listed in the instructions.

PT, Preferred Term; PRR, Proportional Reporting Ratio; ROR, Reporting Odds Ratio.

### 3.2 Meta-analysis

#### 3.2.1 Literature search results

**Figure 2:**
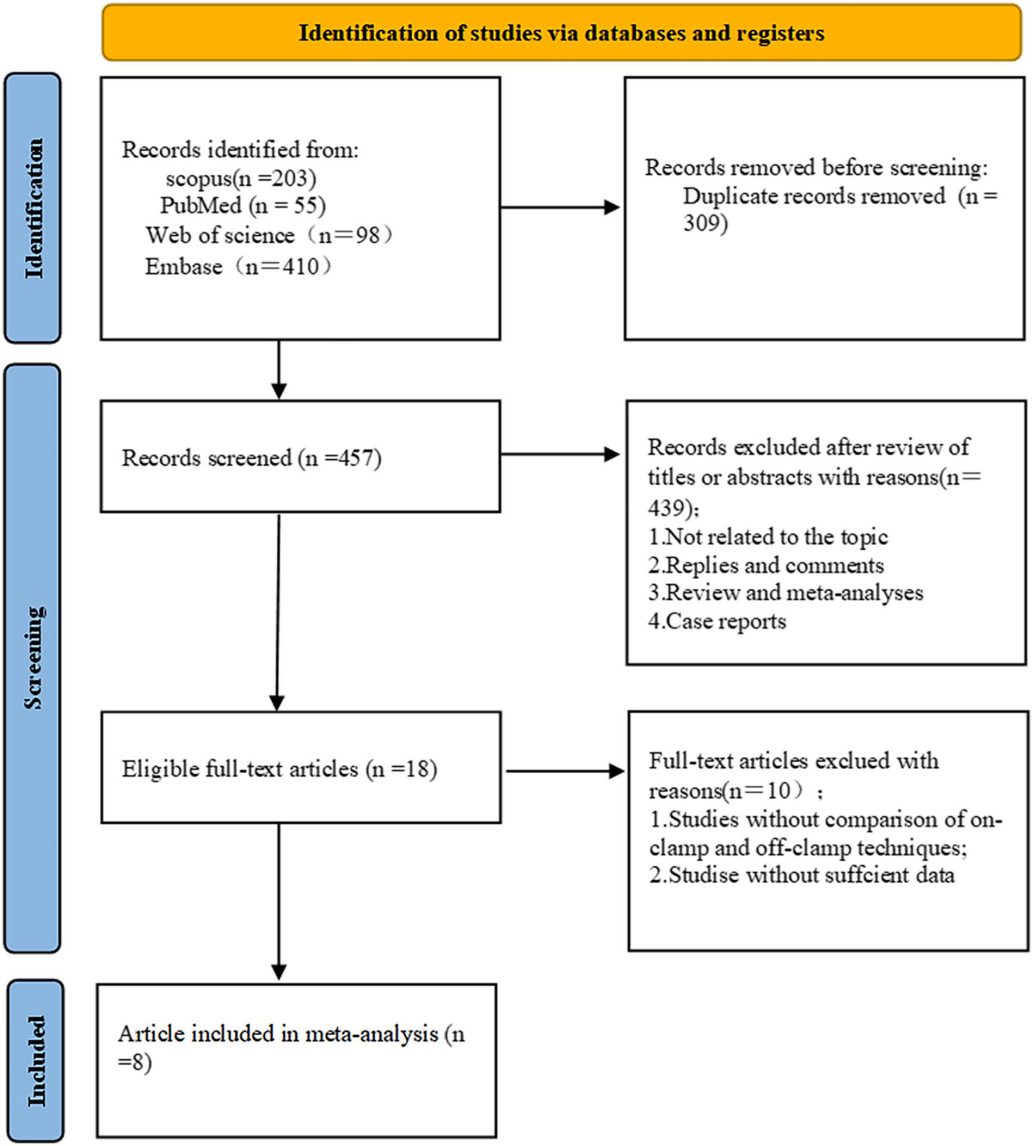
Literature screening flow chart.

Using the search strategy mentioned in the method, a preliminary search obtained 766 articles, 309 duplicates were removed, 391 articles were excluded by reading the title and abstract, 46 reviews were excluded, 2 letters were excluded, and 10 articles without sufficient data or control group were excluded. Finally, 8 articles were included. The results are shown in Figure 2.

#### 3.2.2 Basic characteristics and quality evaluation of included studies

A total of eight observational studies were included in the meta-analysis (15, 35–41). Of these studies, one was conducted in Asia, three in Europe, and four in the United States. The basic characteristics of the included studies are summarized in Table 7. The quality of the included studies was evaluated, and the results are shown in Table 8. The average score of the 8 studies was 7.75, indicating that the quality was high and suitable for meta-analysis.

**Table 7:** Basic characteristics of included studies.

Study title	Author	Data source	Year of publication	Design type	Gender (Male/female)	Age	Course of treatment	Outcome index	Article quality	OR value	Population of intervention group and control group
Antipsychotic drugs and risk of pulmonary embolism	Allenet (41)	‘Premier's Perspective’ database (US clinical and economic database from about 500 acute care hospitals)	2012	Retrospective cohort study	Intervention group: 57% control group: 90%	50–60 years old	Unclear	PE	Medium quality	1.12[1.03,1.22]	Intervention group: 69,975 Control group: 28,272,820
Antipsychotic drugs and risk of idiopathic venous thromboembolism: a nested case-control study using the CPRD	Ishiguro (40)	Clinical Practice Research Datalink	2014	Nested case-control studies	Intervention group: 51% control group: 49%	Ages 20 to 59	14 years	VTE	High quality	1.32[0.71,2.48]	Intervention group: 16 Control group: 41
Antipsychotics and VTE Risk in Postmenopausal Women: Nested Case-Control Study	Wang (46)	Taiwan National Health Insurance Research Database	2016	Nested case-control studies	The subjects were all female	≥50 years old	Unclear	VTE	High quality	4.7[2.18,10.14]	Intervention group: 10 Control group: 34
Antipsychotic drugs and risk of venous thromboembolism: nested case-control study	Parker (42)	QResearch database	2010	Nested case-control studies	Intervention group: 79% control group: 79%	53–77 years old	3 months	VTE	High quality	1.49[1.07,2.08]	Intervention group: 102 Control group: 223
Venous thromboembolism among elderly patients treated with atypical and conventional antipsychotic agents	Liperoti (20)	Systematic Assessment of Geriatric Drug Use via Epidemiology (SAGE) database, which contains data from the Minimum Data Set (MDS)	2005	Retrospective cohort study	Intervention group: 42% control group: 34%	7,762 (13.9%)	6 months	VTE	High quality	1.87[1.06,3.27]	Intervention group: 2,825 Control group: 112,078
						7,762 (13.9%)					
Antipsychotics and VTE: Pharmacovigilance Insights from FDA	Y. Yan (45)	FAERS database from 2004 to 2021	2024	cohort study	Intervention group: 108% control group: 116%	18 −64 years old	Unclear	VTE	Medium quality	2.53 [2.38,2.69]	Intervention group: 1034 Control group: 37,754
A pharmacoepidemiological nested case-control study of risk factors for venous thromboembolism with the focus on diabetes, cancer, socioeconomic group, medications, and comorbidities	Myllylahti (44)	Primary study of FIN-CARING2	2024	Nested case-control studies	Intervention group: 99% control group: 98%	Around 66 years old	16 years	VTE	High quality	1.94 [1.30,2.90]	Intervention group: 52 Control group: 84
Association between antipsychotics and pulmonary embolism: a pharmacovigilance analysis	Huang (43)	Reporting System (FAERS), from the first quarter of 2018 to the first quarter of 2023	2024	cohort study	Intervention group: 102%	20 −89 years old	30 days	PE	High quality	3.98[3.63,4.36]	Intervention group: 461 Control group: 25,891

**Table 8:** NOS quality rating table.

study	Ishiguro 2014	Wang 2016	Parker 2010	Myllylahti 2024
Is the case definition adequate	*	*	*	*
Representativeness of cases	*	*	*	*
Selection of controls	*	*	*	*
Definition of controls	*	*	*	*
Comparability of cases and controls on the basis of the design or analysis	*	**	**	*
Ascertain ment of exposure	*	*	*	*
Same method of ascertainment of exposure	*	*	*	*
Nonresponse rate	*	*	*	*
Total	8	9	9	8
NOS for Assessment of Quality of Included Studies: Cohort Studies				
study	Liperoti 2005	Allent 2012	Huang 2024	Yan 2024
Representativeness of exposed cohort?	*	*	*	*
Selection of the nonexposed cohort?	*	-	*	*
Ascertainment of exposure?	*	*	*	*
Demonstration that outcome of interest was not represent at the start of the study	-	-	*	-
Comparability of Cohort	**	**	*	*
Assessment of outcome	*	*	*	*
Was follow up long enough for outcomes to occur	*	-	*	*
Adequacy of follow up of cohorts	*	*	*	-
Total	8	6	8	6

NOS for Assessment of Quality of Included Studies: Cohort Studies.

#### 3.2.3 Main outcome measures

**Figure 3:**
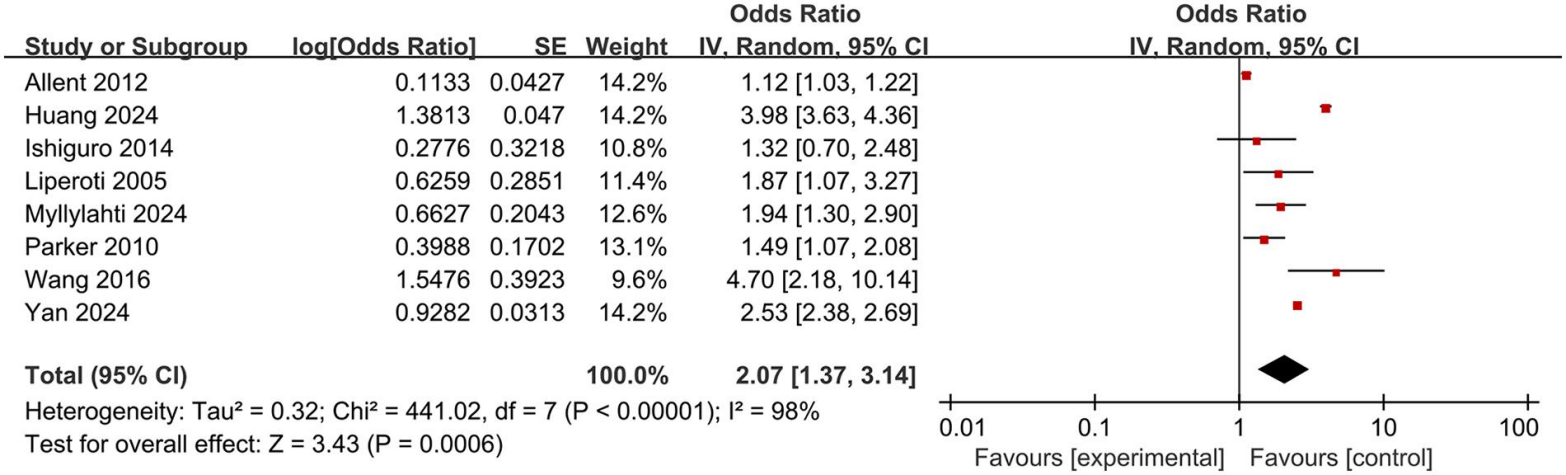
Meta-analysis forest plot of the risk of pulmonary embolism or venous thromboembolism in patients with schizophrenia or mania taking olanzapine.

A total of 8 studies were included in this meta-analysis to explore the association between olanzapine use and the risk of pulmonary embolism and venous thromboembolism. Due to the large statistical heterogeneity (heterogeneity test *P* *<* *0.00001, I^2^* *=* *98%*), a random effects model was used for Meta-analysis, and the combined effect was statistically significant (OR 2.07, 95% CI 1.37–3.14; *P* *=* *0.0006*), indicating that the use of olanzapine in patients with schizophrenia or mania will significantly increase the risk of PE and VTE. The results are shown in Figure 3.

#### 3.2.4 Meta-regression analysis

To explore potential sources of heterogeneity among studies, we performed a meta-regression analysis using disease type, age, race, follow-up duration, study type, and literature quality as covariates. As shown in Table 9, none of the covariates significantly contributed to the heterogeneity of the results.

**Table 9:** Meta-regression analysis.

Covariate	exp(b)	Std.Err.	t	P	95% CI
Disease type	0.951	0.514	−0.09	0.934	0.093–9.730
Age	0.613	0.275	−1.09	0.389	0.089–4.212
Race/ Study type	0.936	0.582	−0.11	0.925	0.065–13.568
Follow-up time	0.566	0.222	−1.45	0.283	0.105–3.052
Literature quality	2.921	1.934	1.62	0.247	0.169–50.456

#### 3.2.5 Subgroup analysis

To evaluate the stability of the results, subgroup analysis was also performed. The subgroup analysis was performed as follows: (1) study type (2) quality of the literature (3) follow-up time (4) race (5) age and (6) disease type. Subgroup analysis showed that the results were relatively stable. Grouping by research type, follow-up time, race, and age did not affect the stability of the results. However, grouping by literature quality and disease type did affect the stability of the results. When grouped according to the quality of the literature, there was no statistically significant difference in the medium-quality literature subgroup (OR 1.68, 95% CI 0.76–3.74; *P* *=* *0.20)*. This may be due to the moderate quality of the included literature. In addition, when grouped by disease type, the subgroup with venous thromboembolism as the disease type (OR 2.04, 95% CI 1.53–2.73; *P* *<* *0.00001*) had a statistically significant difference, indicating that the data support that the use of olanzapine in patients with schizophrenia or mania increases the risk of VTE. There was no significant difference in the subgroup with pulmonary embolism (OR 2.11, 95% CI 0.63–7.31; *P* *=* *0.24*). The reason for the effect may be due to the small number of included literature. The risk of PE in patients was compared with the risk of VTE in patients. Although the OR value of PE was slightly higher than that of VTE, the data were not statistically significant and the heterogeneity of the PE subgroup (*I²* = 100%) was higher than that of the VTE subgroup (*I²* = 73%), indicating that the stability of the results of the PE subgroup was worse. Therefore, overall, the association between olanzapine use and VTE is slightly stronger than that between olanzapine use and PE. In addition, when grouped according to follow-up time, it was found that patients with a follow-up time of more than 1 year had a slightly lower risk (OR 1.73, 95% CI 1.23–2.44; *P* *=* *0.002*), which may be related to metabolism. The results of subgroup analysis are shown in Supplementary Figure S2.

#### 3.2.6 Sensitivity analysis

**Figure 4:**
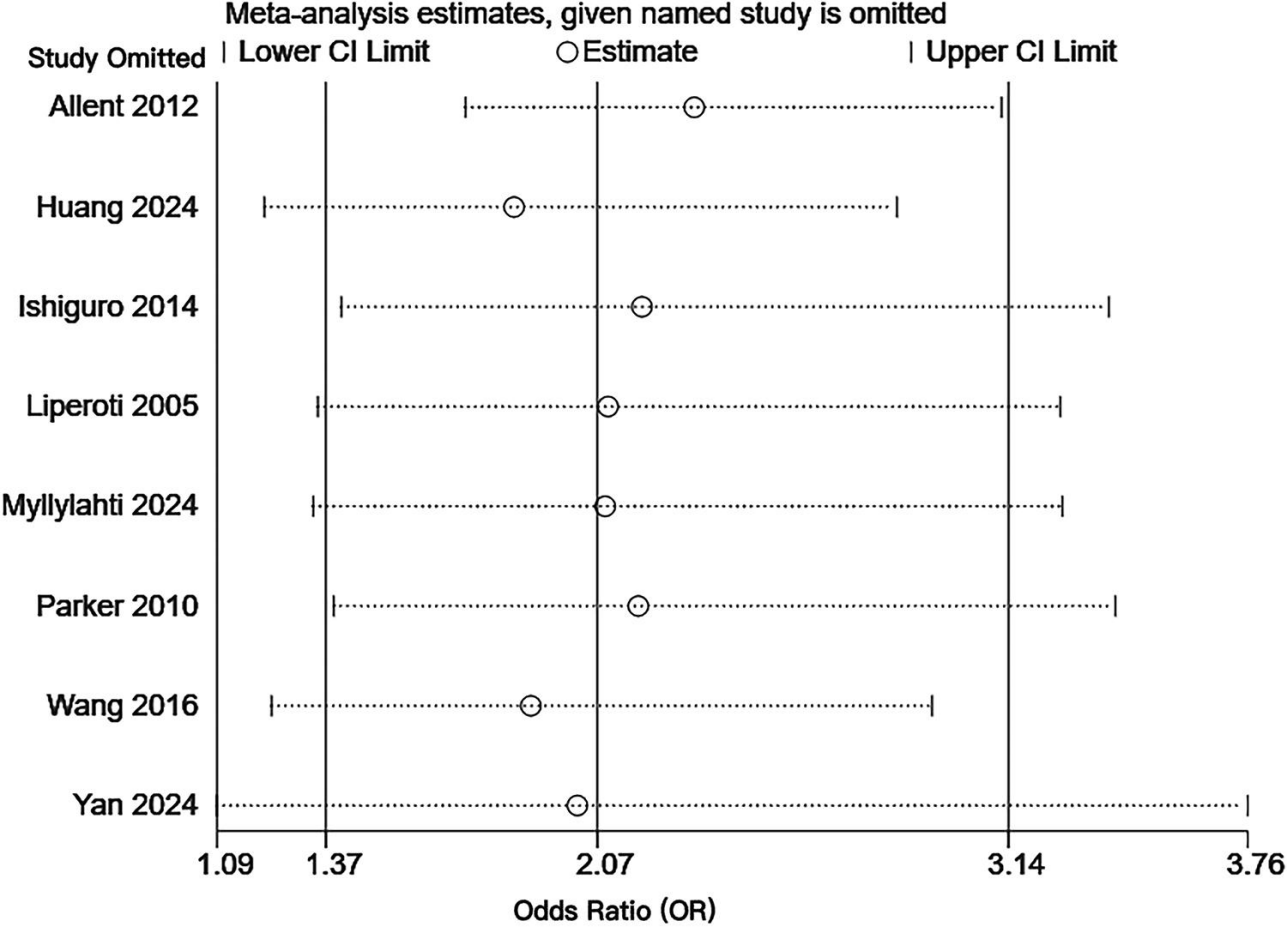
Sensitivity analysis of the risk of pulmonary embolism or venous thromboembolism in patients with schizophrenia or mania taking olanzapine.

Sensitivity analysis was performed by eliminating individual articles one by one. After excluding each individual study in turn, the combined effect size was analyzed separately. It was found that the result range of the OR value of each combined effect size remained at 95%CI (1.37–3.14), the combined result did not change significantly, and the confidence interval of each combined effect size did not cross the ineffectiveness line. The results were statistically significant, and the overall trend of each combined effect size did not change, indicating that the results were relatively stable. The results of the sensitivity analysis are shown in Figure 4. Detailed data of the sensitivity analysis using Stata software are shown in Table 10.

**Table 10:** Sensitivity analysis data.

Study omitted	Estimate	[95% Cl]	*I* ^2^
Allent 2012	2.32,51,377	1.73,15,247	3.12,22,571 94%
Huang 2024	1.85,75,449	1.21,01,998	2.85,11,598 98%
Ishiguro 2014	2.18,94,292	1.40,95,607	3.40,07,762 99%
Liperoti 2005	2.10,14,549	1.34,86,848	3.27,43,848 99%
Myllylahti 2024	2.094001	1.33,66,722	3.28,04,156 99%
Parker 2010	2.17,97,236	1.39,01,326	3.41,77,999 99%
Wang 2016	1.901185	1.22,86,693	2.94,18,042 99%
Yan 2024	2.02,17,985	1.08,68,746	3.760939 99%
Combined	2.07,35,739	1.36,92,355	3.14,02,259 98%

#### 3.2.7 Publication bias

**Figure 5:**
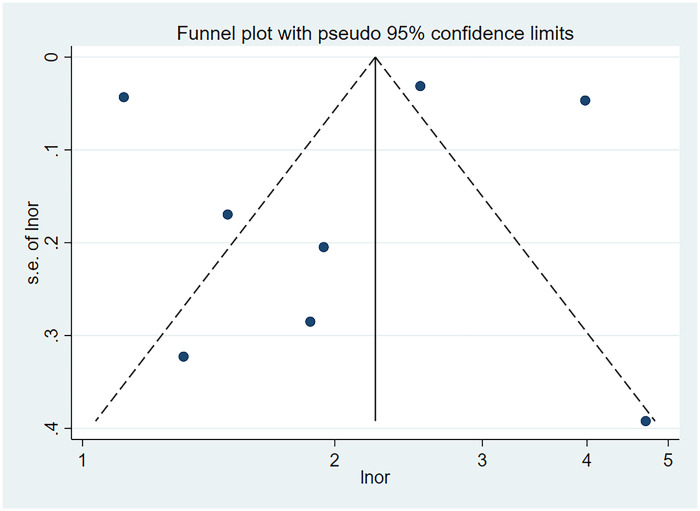
Funnel plot of the risk of pulmonary embolism or venous thromboembolism in patients with schizophrenia or mania taking olanzapine.

**Figure 6:**
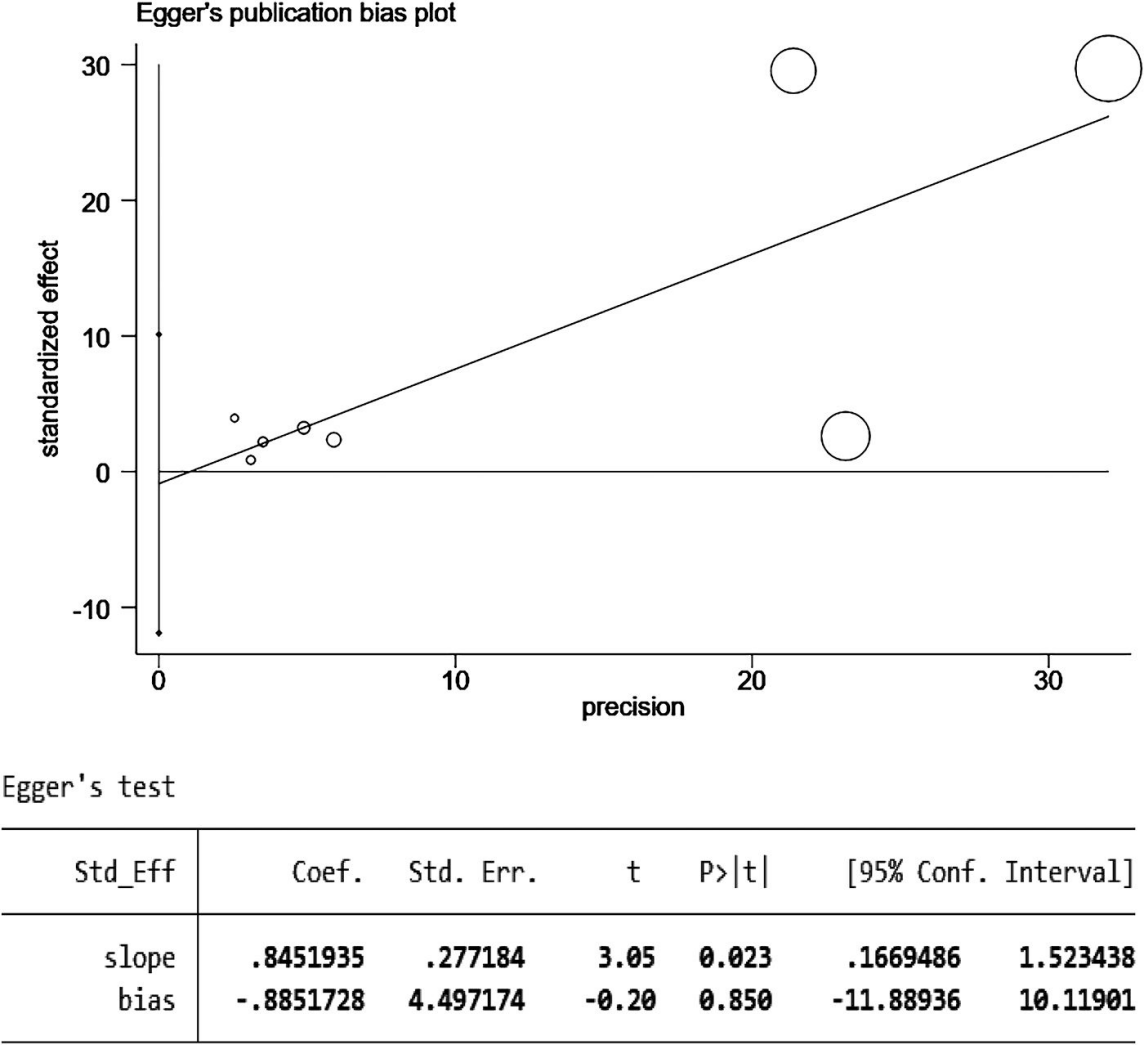
Egger test for the risk of pulmonary embolism or venous thromboembolism in patients with schizophrenia or mania taking olanzapine.

The results of the funnel plot are shown in Figure 5. All studies are distributed in the funnel plot and the scatter points are roughly symmetrical, suggesting that the publication bias of each study is small, indicating that the bias of the literature included in this study is well controlled. In order to ensure the accuracy and reliability of the study, it is necessary to conduct the Egger test. After the Egger test, it was found that *P* = 0.850 > 0.05, indicating that there was no obvious publication bias among the included literature in the study on the correlation between schizophrenia and mania patients using olanzapine and the occurrence of venous thromboembolism and pulmonary embolism. The results are shown in Figure 6.

## 4 Discussion

This study is the first to combine FAERS database signal mining with a systematic meta-analysis of PE and VTE to explore the potential thrombotic adverse events of olanzapine.

FAERS data showed that a total of 55,905 olanzapine-related reports were included in this study. Basic information shows that there are more male patients than female patients (1:0.97), suggesting that male patients should pay more attention to medication risks. Basic information shows that the 18–40 age group (23.9%,95% CI:23.6%–24.2%) and the 41–64 age group (29.2%,95%CI:28.8%–29.6%) are the main reporting populations. Meanwhile, patients aged 65 and above (65–85 years old account for 14.7%,95% CI:14.4%–15.0% and ≥86 years old account for 1.6%,95% CI:1.5%–1.7%) also constitute a significant proportion. The 41–64 age group has the highest proportion and is the core population for olanzapine-related adverse event reports; the risk in this age group deserves special attention. This age group often faces high work pressure, sedentary lifestyles, and other lifestyle problems. Some individuals may already have underlying conditions such as early-stage hypertension and dyslipidemia. Olanzapine-induced metabolic disorders (such as obesity and dyslipidemia) and venous congestion significantly increase the risk of thrombosis. The elderly population over 65 years of age already has a higher risk of thrombosis due to underlying conditions such as hypertension and diabetes, decreased vascular function, and venous congestion. Olanzapine-induced metabolic abnormalities further exacerbate this risk. After comparing the drug instructions, it was found that olanzapine-related pulmonary embolism signals ranked 25th among all adverse events and were not clearly recorded in the adverse reactions in the instructions. In addition, there are 11 ADE signals among the top 50 reported cases that are not included in the olanzapine drug instructions, and the rest include suicidal behavior, electrocardiogram QT interval prolongation, febrile neutropenia, delirium, hyponatremia, attention disorder, tension, abnormal blood prolactin, myocarditis, infectious aspiration pneumonia, and excessive salivation. Therefore, when olanzapine is used clinically to treat schizophrenia, the above-mentioned ADEs should be taken seriously and differentiated from primary diseases. A secondary search revealed that the ROR values of olanzapine for pulmonary embolism and venous embolism were relatively high, namely pulmonary embolism [ROR (95% CI): 2.45 (2.28, 2.64)] and venous embolism [ROR (95% CI): 5.67 (4.49, 7.17)], and venous embolism was also an adverse reaction not clearly recorded in the instructions. Among the 25 SOCs, the top 10 in terms of reporting cases are psychiatric diseases, various neurological diseases, various examinations, various injuries and procedural complications, systemic diseases, and various reactions at the administration site. Among them, the ADE signals of mental illnesses, various nervous system diseases and various examinations mostly overlap with the known pharmacological effects of olanzapine. Therefore, it is necessary to focus on adverse event signals that are not included in the instructions in SOCs such as metabolic and nutritional diseases, cardiac diseases, blood and lymphatic system diseases and respiratory system diseases.

Systematic reviews and meta-analyses demonstrated that patients treated with olanzapine had a significantly increased risk of VTE and PE compared to those not receiving olanzapine (OR 2.07, 95% CI 1.37–3.14; *P* *=* *0.0006*). This finding was consistent with the signal strength observed in the FAERS database, where the reporting odds ratio (ROR) for pulmonary embolism and venous embolism was 2.45 and 5.67, respectively, further supporting an elevated thrombotic risk. These results reinforce the reliability of the FAERS signal detection outcomes. Subgroup analysis indicated that in the medium-quality literature subgroup, the association did not reach statistical significance (OR 1.68, 95% CI 0.76–3.74; *P* *=* *0.20*), possibly due to methodological limitations such as lower study quality and unclear follow-up durations. Furthermore, in the subgroup analysis by disease type, the PE subgroup (OR 2.11, 95% CI 0.61–7.31; *P* *=* *0.24*) did not achieve statistical significance, which may be attributed to the limited number of included studies (*n* = 2) and high heterogeneity (*I²* = 100%), suggesting limited robustness of the results. In contrast, the VTE subgroup included 6 studies with a pooled OR (OR 2.04, 95% CI 1.53–2.73; *P* *<* *0.00001*) and lower heterogeneity than the PE subgroup (*I²* = 73%). This difference in values suggests that the association between olanzapine and VTE may be slightly stronger than that between olanzapine and PE. This may be due to the higher clinical diagnosis rate of VTE and more complete data from included studies, whereas PE presents rapidly, with some cases remaining undiagnosed, leading to insufficient sample size. The above shows that although the overall use of olanzapine is significantly associated with an increased risk of venous thromboembolism and pulmonary embolism, the differences in results between different subgroups suggest that we need to consider the influence of literature quality and disease type when interpreting the conclusions.

However, the interpretation of these results must take into account the potential confounding factors inherent in the nature of the data source observations. First, patients with mental illnesses (such as schizophrenia and bipolar disorder) often have lifestyle-related risks such as sedentary lifestyles, smoking, and poor diet, which can induce venous stasis and a hypercoagulable state (42). Furthermore, mental illness itself is associated with chronic low-grade inflammation and hypothalamic-pituitary-adrenal (HPA) axis dysfunction, and these pathological conditions may independently promote thrombosis (43). Second, olanzapine is used to prevent chemotherapy-induced nausea and vomiting (CINV) in cancer patients, and malignancy, through tumor cell-induced coagulation activation, is a major risk factor for VTE (44). Therefore, we used MedDRA® version 26.0 standardized coding to perform a “malignancy” specific search on olanzapine-related adverse event reports in the FAERS database. The results showed that only 229 reports (0.4%) explicitly documented malignancy. Although the FAERS database contains spontaneously reported cases, and some reports lack complete comorbidity information, the proportion of recorded malignant tumor cases is extremely low, suggesting that this factor is not the primary driver of the observed thrombotic signals. Furthermore, in the inclusion and exclusion criteria for the meta-analysis, we explicitly limited the study population to patients diagnosed with schizophrenia or mania, and excluded studies involving patients with malignant tumors or those using olanzapine due to chemotherapy-related nausea and vomiting (CINV). This rigorous screening ensured that the meta-analysis results came from populations without concomitant malignant tumors, directly supporting the association between olanzapine and thrombotic risk independent of cancer factors. We acknowledge that incomplete comorbidity documentation in the FAERS database may lead to unrecorded malignant cases, which is a potential limitation of this study. Future studies will employ a prospective cohort design, explicitly screening and excluding patients with malignant tumors at enrollment, collecting detailed information such as tumor history and treatment status, and further controlling for this confounding factor through propensity score matching to more accurately quantify the independent effect of olanzapine on thrombosis risk.

This study found that the use of olanzapine significantly increased the risk of PE and VTE; however, it did not explore the underlying mechanisms by which olanzapine induces thrombosis. To address this gap, we conducted a literature review to explore the potential mechanisms through which olanzapine may contribute to the development of PE or VTE. First, olanzapine increases the risk of thrombosis mainly by inducing metabolic syndrome (45). Metabolic syndrome is characterized by obesity, dyslipidemia, insulin resistance (IR), and abnormal blood glucose metabolism. This not only increases the risk of patients developing type 2 diabetes and cardiovascular disease (46), but also accelerates atherosclerosis. Dyslipidemia promotes lipid deposition in the vascular endothelium, leading to inflammation and plaque formation. In rabbit coronary arteries with atherosclerosis, the vasoconstrictive response to serotonin was significantly enhanced. This hyperconstriction was mainly mediated by 5-HT_1_B receptors and led to enhanced intracellular calcium mobilization in smooth muscle cells through pertussis toxin-sensitive signaling pathways. In diseased vessels, the mRNA level of 5-HT_1_B receptors was also significantly upregulated. This excessive vasoconstriction may restrict blood flow, induce angina, and exacerbate vascular occlusion when plaques rupture.Atherosclerotic plaques disrupt endothelial integrity, expose subendothelial collagen, and activate platelet adhesion and aggregation—a key step in arterial thrombosis (47). Furthermore, in order to determine the drug-induced nature of olanzapine itself and exclude the influence of patients with metabolic syndrome, we consulted the Faers database and found that there were only 193 cases (0.3%) of metabolic syndrome patients in the original database. This indicates that the proportion of recorded cases of metabolic syndrome is extremely low, suggesting that this factor is not the main driving factor of the observed thrombotic signals. In addition, metabolic abnormalities caused by olanzapine can also lead to impaired endothelial function and increase the risk of thrombosis (48). Vascular endothelial injury is another key mechanism underlying thrombosis. Studies have shown that olanzapine can induce oxidative stress and inflammatory responses, resulting in endothelial cell damage and dysfunction (49). Olanzapine promotes the inflammatory response in atherosclerosis by disrupting aortic cholesterol homeostasis and the regulation of hepatic lipid metabolism, leading to endothelial dysfunction (50). Moreover, olanzapine-induced oxidative stress increases the production of reactive oxygen species (ROS) and reduces nitric oxide (NO) synthesis, causing vasoconstriction and promoting thrombosis (51). Oxidative stress and inflammation can also dysregulate the vascular endothelial growth factor (VEGF) signaling pathway, leading to increased vascular permeability, endothelial apoptosis, and abnormal angiogenesis (52). Furthermore, recent studies have shown that olanzapine can induce various stress responses in pancreatic *β*-cell vesicles, thereby impairing insulin secretion. In pancreatic *β*-cell lines, olanzapine can reduce insulin secretion at clinically relevant concentrations (64–160 nM). Studies have shown that *β*-cells express multiple dopamine, serotonin, and histamine receptors. Olanzapine may directly inhibit insulin secretion by inhibiting dopamine D_3_ receptors, serotonin 5-HT_2_B and 5-HT_2_C receptors, and histamine H1 receptors. Insulin resistance and hyperinsulinemia can lead to endothelial dysfunction, a pro-inflammatory state, and inhibition of the fibrinolytic system (e.g., elevated plasminogen activator inhibitor-1), thereby creating a vascular environment conducive to thrombosis (53). Finally, subgroup analysis in the current study revealed that patients with a follow-up period longer than one year had a slightly lower risk of thrombosis [OR = 1.73, 95% CI (1.23, 2.44), *P* = 0.31], suggesting that metabolic adaptation or clinical monitoring may partially mitigate the long-term thrombotic risk associated with olanzapine use. However, further investigation is needed to confirm this hypothesis.

Among the second-generation antipsychotics, both olanzapine and clozapine are associated with an increased risk of thrombosis, but the mechanisms and magnitude of risk may differ between the two. Clozapine increases the risk of thrombosis by directly affecting the coagulation system (54), and users of clozapine have a higher risk of venous thromboembolism and pulmonary embolism than users of olanzapine (18). However, compared with other antipsychotics, olanzapine has more significant metabolic side effects (55). A retrospective cohort study of nursing home residents showed that the adjusted hazard ratio (HR) for hospitalization due to VTE in patients using risperidone was 1.98 (95% CI, 1.40–2.78) (15). This indicates a risk level similar to olanzapine. There are also case reports of the same patient experiencing pulmonary embolism after consecutive use of olanzapine and risperidone, suggesting that these two drugs with similar 5-HT2 receptor antagonistic properties may both be associated with this adverse reaction (56).In addition, one study detected an association signal between quetiapine and VTE (ROR = 1.37), suggesting that the risk of quetiapine may be relatively low, but not entirely risk-free, and may vary across different populations (40). Most evidence tends to suggest that aripiprazole carries a lower risk, with some studies suggesting a lower likelihood of pulmonary embolism in patients taking aripiprazole (57).

Studies have also shown that other antipsychotic drugs (APS) may induce thrombosis, with platelet activation playing a more prominent role in the underlying mechanism. Dai et al. reported that the use of APS was associated with the risk of VTE and PE (58). First, antipsychotics can enhance platelet aggregation, which is one of the key factors leading to thrombosis (59). At the same time, antipsychotic drugs are also associated with increased platelet activation markers, further increasing the risk of thrombosis (60). Secondly, 5-HT is an important mediator of platelet activation and aggregation. Antipsychotic drugs regulate the 5-HT system to affect platelet function, indirectly affecting platelet activation and aggregation (61). In addition, the metabolites of antipsychotic drugs may also indirectly affect platelet function by inhibiting platelet aggregation-related enzymes (cyclooxygenase) or changing the fluidity of platelet membranes (62). Moreover, long-term use of antipsychotic drugs may lead to drug accumulation, further exacerbating the effects on platelet function (62).

In addition, according to the FAERS database, olanzapine may also cause other unrecorded adverse reactions. Febrile neutropenia: Immune-mediated neutrophil damage and cytokine dysregulation lead to fever (63–65). Hyponatremia: Central dopamine D_2_ and 5-HT_2_Areceptors antagonism interferes with the regulation of antidiuretic hormone (ADH) secretion and drug-induced polydipsia, leading to hyponatremia (66–69). Myocarditis: Olanzapine antagonizes 5-HT_2_A receptors, histamine H1 receptors, and muscarinic M3 receptors. It can reduce coronary blood flow, inhibit cardiac ATP production, and change the phosphorylation level of acetyl-CoA carboxylase, directly affecting myocardial energy metabolism. The pro-inflammatory response caused by olanzapine may be involved in the occurrence of myocarditis (70–74). Infectious aspiration pneumonia: The significant anticholinergic and sedative effects of high-dose olanzapine inhibit the swallowing reflex and throat coordination, increasing the risk of aspiration and aspiration pneumonia. Chronic inflammation associated with metabolic syndrome may also weaken lung defenses (74–79). Therefore, potential adverse events such as cardiovascular, blood, and respiratory system events need to be monitored during clinical use of olanzapine to guide safer and more individualized drug use strategies.

This study has several limitations that should be acknowledged. First, the FAERS database is based on spontaneous reporting, which may lead to under-reporting, duplicate submissions, and potential inaccuracies in the data, thereby introducing reporting bias into the analysis (80). Second, since the core data of the FAERS database are predominantly derived from the healthcare system in the United States, the generalizability of the findings to other countries or regions with differing healthcare policies, population characteristics, or clinical practices may be limited, and thus their applicability should be carefully evaluated in light of regional differences. Third, the detection of ADE signals indicates only a statistical association, and further clinical investigations are required to establish a causal relationship supported by biological mechanisms. Despite these limitations, this study provides valuable insights into pharmacovigilance signal detection and highlights the importance of enhanced post-marketing surveillance. Moreover, the meta-analysis exhibited substantial heterogeneity, primarily attributable to variations in study design, exposure definitions, and population characteristics. The number of eligible studies included was relatively limited, especially for pulmonary embolism, indicating a need for future large-scale, high-quality research to further validate these findings. In particular, more robust cohort and case-control studies focusing on pulmonary embolism are needed to strengthen the evidence base. Additionally, observational studies such as retrospective cohorts and case-control designs are inherently susceptible to residual confounding from variables including concomitant medications, lifestyle factors, age, sex, environmental exposures, dietary habits, and daily routines. Although most of the included studies attempted to control for such confounders, the possibility of residual bias cannot be completely excluded. Furthermore, the relationship between olanzapine dosage and thrombotic risk was not comprehensively examined in the included studies, limiting the clinical interpretability and guidance regarding dose-related risk.

Based on the existing evidence, clinicians need to be alert to the risk of thrombosis during olanzapine treatment, especially for high-risk groups such as elderly patients, obese patients or those with venous congestion. It is recommended to assess the patient's baseline thrombotic risk before medication, strengthen monitoring during treatment, and consider preventive measures. For patients taking long-term medication, metabolic indicators need to be assessed regularly to intervene in related side effects at an early stage. Future research can conduct prospective cohort studies to control confounding factors and clarify the dose-effect relationship. Explore the role of biomarkers (e.g., inflammatory factors, coagulation markers) in risk prediction. To evaluate the effectiveness of prophylactic interventions (such as risk-assessed anticoagulation therapy) in reducing thrombotic events in certain high-risk olanzapine users.

## 5 Conclusion

Based on the results of signal detection from the FAERS database, as well as findings from a systematic review and meta-analysis, this study confirmed that olanzapine is significantly associated with an increased risk of PE and VTE. The systematic review and meta-analysis demonstrated that, compared with non-users, olanzapine users had a significantly elevated combined risk ratio for PE and VTE (OR 2.07, 95% CI 1.37–3.14; *P* *=* *0.0006*). The underlying mechanisms by which olanzapine may induce thrombotic events could involve metabolic disturbances, enhanced platelet aggregation, and impaired venous return. This risk is more pronounced in high-risk groups, including core users aged 41–64 (29.2%,95% CI:28.8%–29.6% of the total reported cases), the elderly aged 65 and above (16.3%,95% CI:16.0%–16.6%), obese patients, and patients with venous stasis. Given the widespread use of olanzapine, clinicians should maintain a high level of vigilance for thromboembolic adverse events in patients receiving this medication, particularly those at elevated risk, and should consider appropriate preventive and interventional strategies.

## Data Availability

The original contributions presented in the study are included in the article/Supplementary Material, further inquiries can be directed to the corresponding author/s.
