# Supplementary material for: Thromboembolic events with olanzapine: a systematic review integrating meta-analysis and FAERS database
Source: Front Cardiovasc Med. 2026 Feb 11;13:1710507. doi: 10.3389/fcvm.2026.1710507 (PMC12932513; doi:10.3389/fcvm.2026.1710507)
Supplement: Supplementary file 1 [file Datasheet1.docx]

**Thromboembolic events with olanzapine: a systematic review integrating meta-analysis and FAERS database**


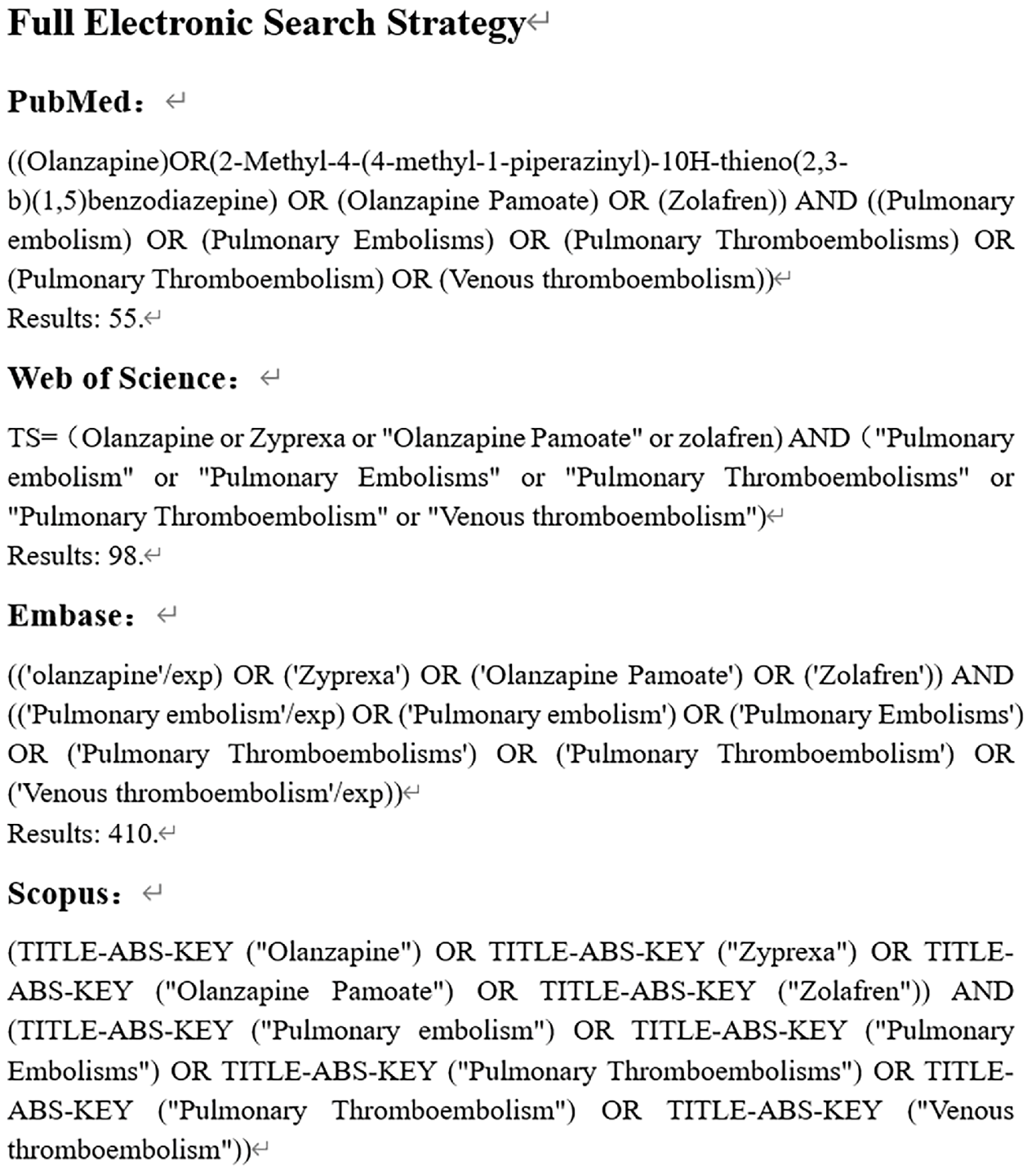


**Figure.S1** Olanzapine and pulmonary embolism and venous thromboembolism Full search strategy


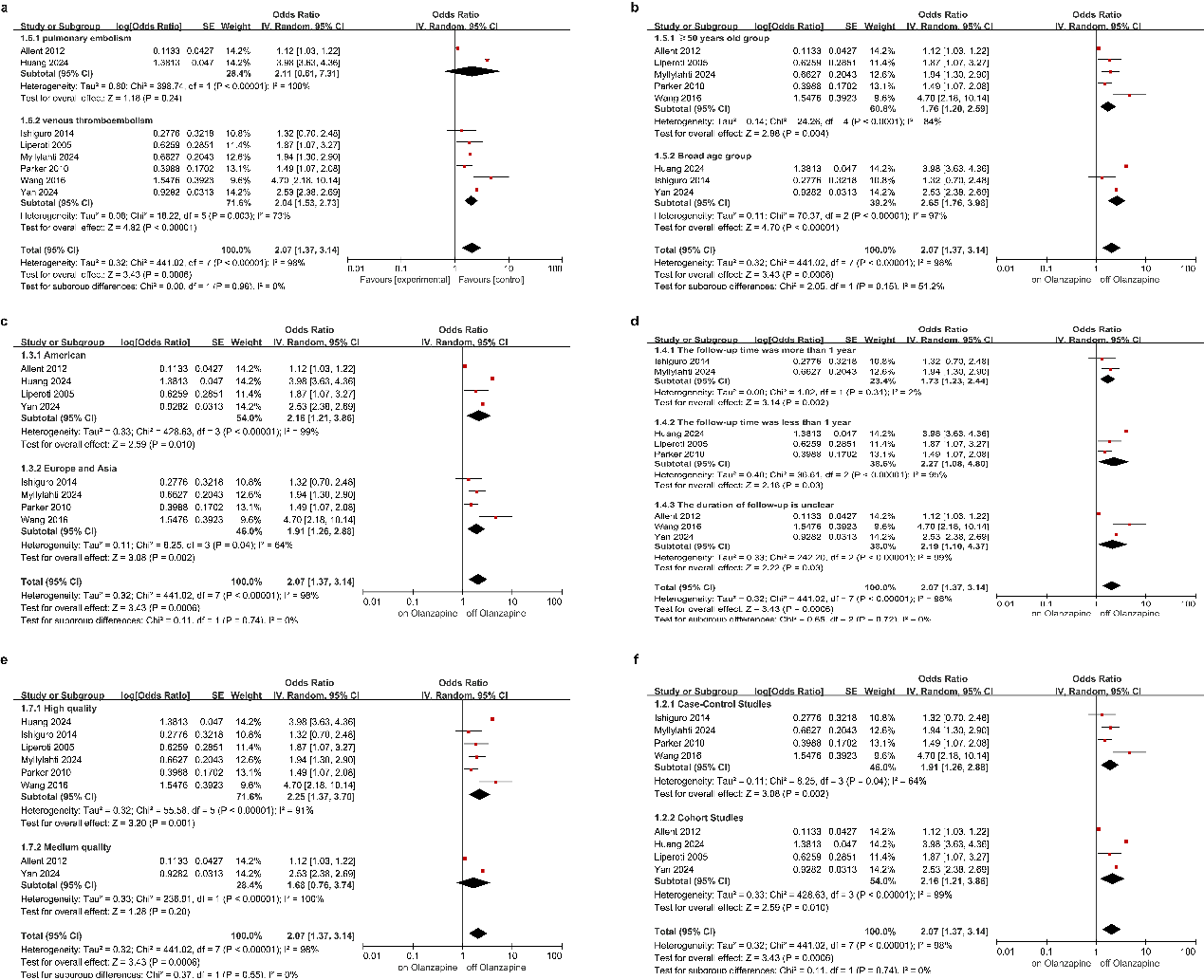


**Figure.S2** Subgroup Analysis of the Association between Olanzapine and Pulmonary Embolism and VTE

**Figure.S2a** Subgroup Analysis Based on Disease Type **Figure.S2b** Subgroup Analysis Based on Age in the Original Study **Figure.S2c** Subgroup Analysis Based on Race in the Original Study **Figure.S2d** Subgroup Analysis Based on Follow-up Duration in the Original Study **Figure.S2e** Subgroup Analysis Based on Study Quality in the Original Study**Figure.S2f** Subgroup Analysis Based on Study Type in the Original Study
